# The direct effect of exercise on the mental health of scientific and technological professionals and the mediating effects of stress, resilience, and social support

**DOI:** 10.3389/fpubh.2023.1074418

**Published:** 2023-03-23

**Authors:** Kai Wu, Shengnan Wang, Tengyun Ding, Yongxin Li

**Affiliations:** ^1^Institute of Psychology and Behavior, Henan University, Kaifeng, China; ^2^Institute of International Education, Henan University of Animal Husbandry and Economy, Zhengzhou, China

**Keywords:** exercise, mental health, stress, resilience, social support, scientific and technology workers

## Abstract

**Background:**

High working pressure is one of the main causes of occupational sub-health problems in scientific and technological professionals. With the development of exercise psychology, an increasing number of scholars have begun to focus on the influence of exercise on mental health. However, a limited number of scholars have investigated the effects of exercise on mental health and related mechanisms among scientific and technological professionals. Thus, this study aimed to investigate the relationship between exercise and the mental health of scientific and technological professionals and the mediating roles of stress, resilience, and social support.

**Methods:**

The respondents in this study were recruited using snowball sampling techniques and finally collected a total of 1,248 valid responses. Questionnaires were distributed through “www.wjx.cn (An online questionnaire website in China, which provides similar functions with Amazon MTurk)” in Henan province from November to December 2021. Participants were asked to complete the Positive Mental Health Scale, the stress subscale of the Depression Anxiety and Stress Scale (21 items), the Connor–Davidson Resilience Scale, and the Perceived Social Support Scale. A total of 1,248 valid responses were obtained.

**Results:**

The mean exercise time of males was longer than that of females (*t* = 3.65, *p* < 0.01). Exercise and mental health were significantly associated with differences in age (*F* = −8.57, *F* = −4.66, *p* < 0.01) and educational background (*F* = 12.86, *F* = 7.14, *p* < 0.01). There were significant correlations among exercise, mental health, stress, resilience, and social support (*p* < 0.01). The direct effects of exercise on mental health were significant (β = 0.271, *t* = 9.577, *p* < 0.001), and the mediating effects of stress, resilience, and social support were also significant (χ^2^/df = 4.72, CFI = 0.940, TLI = 0.935, SRMR = 0.048, RMSEA = 0.055).

**Conclusion:**

This study explored the effects of exercise on mental health and related mechanisms among scientific and technological professionals, which is beneficial to providing effective suggestions for managing and preventing the mental health of scientific and technological professionals. Future research should include a wider range of participants and adopt a longitudinal follow-up design to more deeply investigate the relationship between exercise and the mental health of scientific and technological professionals.

## 1. Introduction

Scientific and technological workers master systematic knowledge of relevant majors in the field of natural sciences. They are engaged in the research, development, dissemination, popularization, application, and auxiliary work of science and technology, and they are specialized in the management of science and technology. They mainly include engineering technicians, health technicians, agricultural technicians, scientific researchers, and teaching staff (1). As the main individuals responsible for scientific and technological innovation in China, they solve relevant developmental problems in their respective fields. Working overtime, sitting for long periods, exercising too little, and overworking have become normal phenomena at work (2). Surveys have shown that Chinese scientific and technological professionals work long hours with high intensity and high pressure and have poor mental health (3).

Among the various factors affecting individual mental health, the positive effects of physical activity have received increasing attention and recognition (4). Exercise can eliminate fatigue, strengthen the body, enhance immunity, and provide many benefits to physical health. Moreover, many studies have shown that physical exercise is significantly associated with mental health (5–8). For example, the British government consistently recommends regular physical activity to maintain and improve physical and mental health (9). As a group engaged in mental work for long periods, the specific situation of the way, type, and time of participating in exercise remains unclear in scientific and technology workers. Furthermore, the mechanism by which exercise affects mental health has not been empirically studied sufficiently.

Researchers have shown that individuals can develop positive psychological qualities such as self-efficacy, confidence, and resilience when participating in physical activities (4). Exercise could strengthen immunity, reduce stress hormone levels as well as muscle tension, and could produce an anti-anxiety effect (7). Insufficient exercise, and engaging in avoidance behaviors, all of which can exacerbate or increase the risk of chronic health conditions (8). Exercise can both reduce negative symptoms and increase positive symptoms of mental health (10). In fact, when people engage in less physical activity, they experience an increase in depression, anxiety, and stress (8). Some scholars have posited that physical exercise can directly improve individual mental health and play a mediating role by enhancing self-efficacy and indirectly affecting individual mental health (5). This suggests that there is more than one pathway between exercise and mental health and that it warrants additional exploration. Thus, this study investigated the potential mediating variables of stress, psychological resilience, and perceived social support in the relationship between exercise and the mental health of scientific and technological workers.

### 1.1. Exercise and mental health

Numerous studies have been conducted on the relationship between exercise and mental health. Weinstein et al. (11) comprehensively analyzed 19 studies on the influence of exercise withdrawal on mental health and showed that exercise withdrawal continuously led to an increase in depressive symptoms and anxiety, and experimentally-controlled exercise withdrawal had adverse effects on mental health. Some researchers have also noted that physical activity may help prevent depression, anxiety, and stress disorders, suggesting that it should be used as an auxiliary part of the treatment of psychological problems (12). Moreover, in a large representative sample of 11,110 European adolescents, researchers found that physical activity levels and participation in physical activity tended to predict higher levels of well-being (13). Fluetsch et al. (14) further posited that exercise is a means of improving mental health and happiness.

In China, scholars have widely confirmed that exercise promotes mental health. Some studies have found that even during the COVID-19 pandemic, physical exercise promoted individual mental health (15). There is sufficient evidence to confirm that physical exercise has a positive impact on mental health and well-being both among students and the elderly (4, 5, 16). Moreover, many studies have shown that exercise can effectively reduce patients' symptoms of depression and anxiety, improve life satisfaction, alleviate cognitive decline, and promote mental health (10). Based on the existing research, we developed the following hypothesis:

***Hypothesis 1:** Exercise will have a positive effect on the mental health of scientific and technological professionals*.

### 1.2. Mediation effects of stress

Stress is a physiological and emotional response to threatening and disturbing events. Scholars have found that long-term exposure to stress may result in serious physical and psychological problems (17). When individuals are under high pressure, their physical and mental health is compromised (18). Studies in the field of mental health have shown that both short- and long-term physical exercise can alleviate the negative emotional state caused by stress (19) and help individuals overcome stress, serving as a protective mechanism against the impact of negative stress on health (17). According to Lazarus' pressure interaction model, the stress of science and technology professionals is based on the cognitive explanation of the working environment, and the exercise environment is conducive to the individual's physical and mental relaxation (20). Additionally, according to the American Heart Association, physical exercise can improve the physical and mental quality of life, thereby reducing overall negative stress (17). Therefore, we developed the following mediation model hypothesis:

***Hypothesis 2:** Stress will have an indirect negative effect on the relationship between exercise and the mental health of scientific and technological professionals*.

### 1.3. Mediation effects of resilience

Resilience plays an important role in helping individuals obtain mental health and reduce negative factors. Resilience is a personality trait that can provide relatively stable individual mental health protection (21). Researchers have found that physical exercise can increase the resilience of individuals facing pressure and reduce the incidence of mental illnesses (22). The formation and development of individual resilience benefits from the positive effects of protective factors and is also influenced by negative factors. According to the resiliency model, the presence of biopsychospiritual homeostasis within an individual is influenced by adversity, life events, and protective factors (23). Physical exercise is a protective factor that promotes the development of individual resilience, which helps individuals enhance their control over their bodies, improve their sense of exercise self-efficacy, and improve their sense of control and value. Resilience is important internal resource for reducing individual risk factors, which is more conducive to improving psychological resilience levels (16). Therefore, we formed the following mediation model hypothesis:

***Hypothesis 3:** Resilience will have an indirect positive effect on the relationship between exercise and the mental health of scientific and technological professionals*.

### 1.4. Mediation effects of social support

Social support refers to material or psychological support that individuals can obtain from others through social contact (24). Numerous studies in exercise psychology have shown that regular participation in physical activities can not only increase social networks but are also a useful way to enhance social support (25). Sports are usually performed by several people at the same time. The establishment and maintenance of relationships in sports not only promotes the persistence of sports behaviors but also provides various forms of social support to help individuals solve various psychological and behavioral problems and promote mental health (10). Studies have also verified the social interaction hypothesis, which holds that physical exercise, as a social activity, helps to increase interpersonal communication and enhance individuals' social support, and the enhancement of social support contributes to the improvement of mental health (24). Therefore, we formed the following mediation model hypothesis:

***Hypothesis 4:** Social support will have an indirect positive effect on the relationship between exercise and the mental health of scientific and technological professionals*.

**Figure 1:**
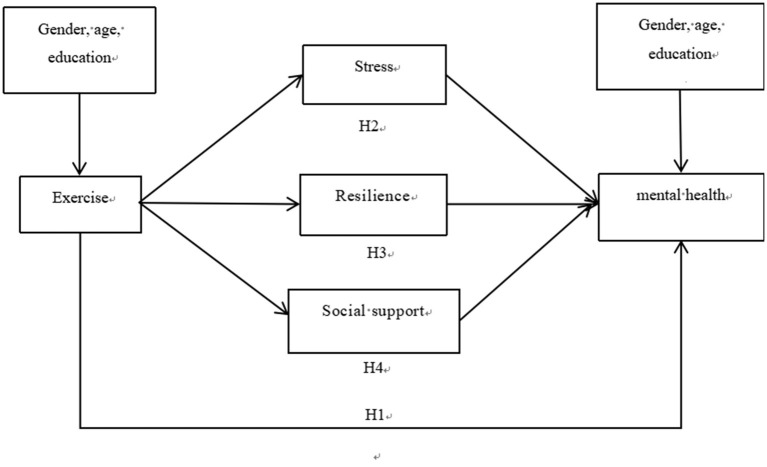
Hypothesized conceptual model.

In sum, this study aimed to explore the effect of exercise on mental health and its mechanisms among scientific and technological professionals in Henan Province, China. The hypothesized model is illustrated in Figure 1.

## 2. Materials and methods

### 2.1. Participants

This study used convenience sampling. The participants were recruited from the cities of Henan Province, in which there are various industries. Before the investigation, all participants were informed of the purpose of the research, relevant precautions for filling out the questionnaire, and the principle of confidentiality. After obtaining consent, data were collected through “www.wjx.cn (An online questionnaire website in China, which provides similar functions with Amazon MTurk)” from November to December 2021. A total of 1248 questionnaires were distributed. After excluding five questionnaires with missing values, 1243 questionnaires were obtained (effective response rate of 99.6%), well beyond the structural equation modeling standard of at least 10:1 or 15:1 samples and observed variables (26). Participants included 634 males (51%) and 609 females (49%) with an average age of 39.68 ± 7.93 years (range, 19–64 years); 981 (78.9%) participants were 45 years old and below, and 261 (21.1%) were 46 years old and above; 599 (48.2%) participants had a doctoral degree, 405 (32.6%) had a master's degree, and 239 (19.2%) had a bachelor's degree or below. The study design was approved by Henan University, and the participants were approved by the Ethical Review Board of the Institution of Psychology and Behavior, and the number of ethical approval is 20211015002.

### 2.2. Measures

#### 2.2.1. Mental health

Mental health was assessed using the Positive Mental Health Scale developed by Lukat et al. (27). The scale is an easy-to-use tool to assess positive mental health and measure the internal and external factors of positive mental health. The scale has a single factor structure and contains nine items measured on a 4-point Likert scale ranging from 1 (incorrect) to 4 (correct). The higher the score, the higher is the level of positive mental health. In various groups, the PMH-Scale showed a unidimensional structure, good test–retest reliability, internal consistency, convergent and discriminant validity (27). For this scale, the Cronbach's alpha coefficient was 0.95.

#### 2.2.2. Stress

Stress was assessed using the stress sub-scale of the Depression Anxiety and Stress Scale (21 items; DASS-21), which is is a simplified version of the DASS (28). The scale includes three subscales of depression, anxiety, and stress, with seven items each. Studies have shown that the DASS-21 has the same stable factor structure and good reliability and validity as the full version of the DASS, and it is more suitable for rapid screening in scientific research and clinical practice. Items describe a person's recent (“in the past week”) negative emotional experiences or corresponding physiological reactions. The 21 items are measured on a 4-point Likert scale ranging from 0 (not at all agree) to 3 (completely agree). The higher the score, the more intense the negative emotional experience. Composite reliability of the stress sub-scale was 0.76, which has stable psychometric properties (28). In this study, the Cronbach's alpha coefficient for the stress sub-scale was 0.93.

#### 2.2.3. Resilience

Resilience was assessed using a simplified version of the Connor–Davidson Resilience Scale (CD-RISC). The simplified version was developed by Campbell-Stlls and Stein (29), and the Chinese version was translated and revised by Li et al. (30). The scale includes 10 items measured on a 5-point Likert scale ranging from 1 (never) to 5 (almost always). The total score is calculated by adding the scores for each item. The higher the total score, the better the resilience. Confirmatory factor analysis showed that the model fit of the mental toughness scale was good: χ^2^/df = 1.87, TLI = 0.935, CFI = 0.918, SRMR = 0.036, RMSEA = 0.069, indicating that the scale had good structural validity (30). For this scale, the Cronbach's alpha coefficient was 0.95.

#### 2.2.4. Social support

Social support was assessed using the Chinese version of the Perceived Social Support Scale (PSSS) (31). The scale includes three dimensions: family support (3, 4, 8, 11), friend support (6, 7, 9, 12), and other support (e.g., leadership, colleagues) (1, 2, 5, 10). The 12 items are measured on a 7-point Likert scale ranging from 1 (strongly disagree) to 7 (strongly agree). The total score is aggregated from all items to reflect the total level of perceived social support. We slightly modified the presentation of the “other support” dimension. For example, in the first item, “When I have a problem, some people (leaders, colleagues, relatives) will come to my side” was changed to “when I have a problem, leaders or colleagues will come to my side.” For this scale, the Cronbach's alpha coefficient was 0.94.

### 2.3. Data analysis

SPSS 26.0, AMOS24.0, and the PROCESS plug-in were used to test hypotheses. First, SPSS 26.0 was used to analyze the demographic differences in exercise and mental health among scientific and technological professionals by *T*-test and analysis of variance. Second, Pearson's correlation analysis was performed to test the correlations between variables. Third, we tested the postulated hypotheses using PROCESS and AMOS 24.0 by structural equation modeling and linear regression analysis. Specifically, we tested the path coefficients about hypotheses by AMOS 24.0, and calculated the percentage of mediating effect by PROCESS.

## 3. Results

### 3.1. Demographic characteristics

Table 1 presents the demographic, exercise, and mental health information of participants. The results indicated a significant difference in the exercise scores (*t* = 3.65, *P* < 0.01) between men and women; compared to women, men exercised longer. Additionally, there were significant differences in exercise time (*t* = −8.57, *P* < 0.01) and mental health (*t* = −4.66, *P* < 0.01) by age, with the scores of those aged 46 and above significantly higher than those aged 45 and below. We also found significant differences in exercise time (*F* = 12.86, *P* < 0.01) and mental health (*F* = 7.14, *P* < 0.01) by education; participants with doctor's or master's degrees had lower scores than those with the highest degree of bachelor's.

**Table 1:** Descriptions among demographic characteristics.

**Variables**	**Categories**	**Exercise time**		**Mental health**	
		***x*** ±***s***	***t*** **/** ***F***	***x*** ±***s***	***t*** **/** ***F***
Gender	Male (*n* = 634)	1.93 ± 0.89	3.65^**^	3.02 ± 0.67	0.51
	Female (*n* = 609)	1.75 ± 0.83		3.00 ± 0.68	
Age	45 years old and below (*n* = 981)	1.72 ± 0.81	−8.57^**^	2.96 ± 0.67	−4.66^**^
	46 years old and above (*n* = 262)	2.27 ± 0.94		3.18 ± 0.65	
Education	Doctorate (*n* = 599)	1.75 ± 0.83	12.86^**^	2.96 ± 0.67	7.14^**^
	Master's (*n* = 405)	1.83 ± 0.88		3.00 ± 0.68	
	Bachelor's or below (*n* = 239)	2.08 ± 0.91		3.15 ± 0.64	

LSD *post hoc* test on education: Doctor, Master < Bachelor and below.

* *P* < 0.05,

** *P* < 0.01.

### 3.2. Correlations

Pearson's correlation analysis was performed to test the correlations between variables. The results of the correlation analysis are presented in Table 2. Exercise time was positively correlated with mental health, resilience, and social support and negatively correlated with stress (all *P* < 0.01). Resilience and social support were positively correlated with mental health, whereas stress was negatively correlated with mental health (all *P* < 0.01).

**Table 2:** Correlation of research variables.

**Variables**	**M ±SD**	**1**	**2**	**3**	**4**	**5**	**6**	**7**
1. Gender	1.49 ± 0.50	1						
2. Age	39.68 ± 7.93	−0.09^**^	1					
3. Education	1.71 ± 0.77	0.12^**^	0.29^**^	1				
4. Exercise time	1.84 ± 0.87	−0.10^**^	0.27^**^	0.14^**^	1			
5. Mental health	3.01 ± 0.67	−0.02	0.10^**^	0.10^**^	0.29^**^	1		
6. Stress	2.09 ± 1.44	−0.07^*^	0.05	−0.09^**^	−0.23^**^	−0.50^**^	1	
7. Resilience	3.79 ± 0.72	−0.09^**^	0.11^**^	0.09^**^	0.21^**^	0.56^**^	−0.36^**^	1
8. Social support	4.90 ± 1.12	−0.05	−0.04	0.07^**^	0.17^**^	0.51^**^	−0.28^**^	0.47^**^

Gender: male = 1, female = 2; Education: doctor = 1; master's degree = 2; bachelor's degree and below = 3. Exercise time: almost no exercise behavior = 1; exercise for 0–30 min a day = 2; exercise for 30–60 min a day = 3; exercise for at least 60 min/day = 4.

* *P* < 0.05,

** *P* < 0.01.

### 3.3. Mediation effects of stress

**Figure 2:**
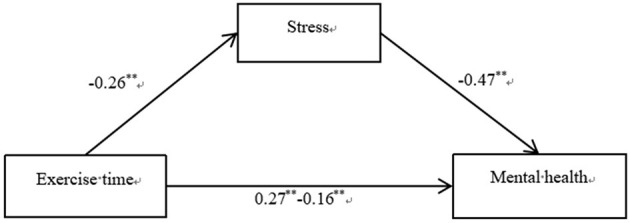
Results of the mediation effect of stress.

The results showed that exercise time had a significant predictive effect on mental health (β = 0.271, *t* = 9.577, *P* < 0.001), and the predictive effect of exercise time on mental health was still significant after adding mediating variables (β = 0.156, *t* = 6.068, *P* < 0.001). Exercise time had a significant predictive effect on stress (β = −0.247, *t* = −8.646, *P* < 0.001), and stress had a significant predictive effect on mental health (β = −0.464, *t* = −18.682, *P* < 0.001). All standardized path coefficients were significant, indicating that exercise time predicted mental health directly and through the mediating effect of stress. The direct (0.121) and mediating (0.089) effects accounted for 57.65% and 42.35% of the total effect (0.210), respectively (Figure 2). Therefore, Hypotheses 1 and 2 were supported.

### 3.4. Mediation effects of resilience

**Figure 3:**
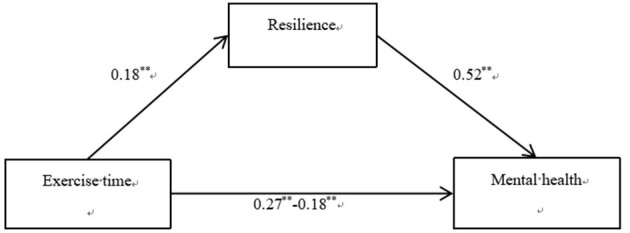
Results of the mediation effect of resilience.

The results showed that exercise time had a significant predictive effect on mental health (β = 0.271, *t* = 9.577, *P* < 0.001), and the predictive effect of exercise time on mental health was still significant after adding mediating variables (β = 0.176, *t* = 7.232, *P* < 0.001). Exercise time had a significant predictive effect on resilience (β = 0.182, *t* = 6.302, *P* < 0.001), and resilience had a significant predictive effect on mental health (β = 0.523, *t* = 22.131, *P* < 0.001). All standardized path coefficients were significant, indicating that exercise time predicted mental health directly and through the mediating effect of resilience. The direct (0.136) and mediating (0.074) effects accounted for 64.95% and 35.05% of the total effect (0.210), respectively (Figure 3). Therefore, Hypotheses 1 and 3 were supported.

### 3.5. Mediation effects of social support

**Figure 4:**
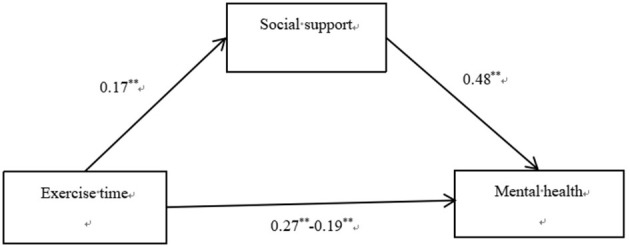
Results of the mediation effect of social support.

The results showed that exercise time had a significant predictive effect on mental health (β = 0.271, *t* = 9.577, *P* < 0.001), and the predictive effect of exercise time on mental health was still significant after adding mediating variables (β = 0.187, *t* = 7.507, *P* < 0.001). Exercise time had a significant predictive effect on resilience (β = 0.174, *t* = 5.994, *P* < 0.001), and resilience had a significant predictive effect on mental health (β = 0.476, *t* = 19.715, *P* < 0.001). All standardized path coefficients were significant, indicating that exercise time predicted mental health directly and through the mediating effect of resilience. The direct (0.146) and mediating (0.064) effects accounted for 69.38% and 30.62% of the total effect (0.210), respectively (Figure 4). Therefore, Hypotheses 1 and 4 were supported.

### 3.6. Mediation effects of stress, resilience, and social support

**Figure 5:**
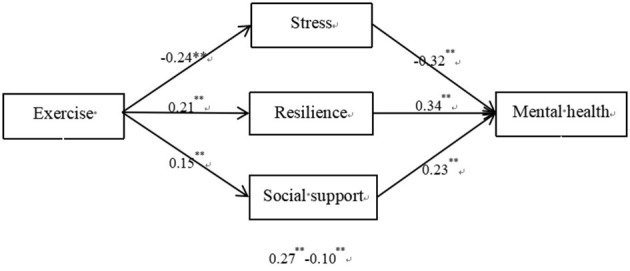
Results of the mediation effect of stress, resilience, and social support.

To further explore the mediating effects of stress, resilience, and social support on the relationship between exercise time and mental health, AMOS was used to establish a structural equation model to further test the mediating effect. The results are shown in Figure 5. For simplicity, the control variables and residual term are not presented. The fit index met the following standards: χ^2^/*df* = 4.72, CFI = 0.940, TLI = 0.935, SRMR = 0.048, and RMSEA = 0.055.

The results showed that the mediating effect included three indirect effects: indirect effect 1, exercise time → stress → mental health, the mediating effect of stress accounted for 26.80% of the total effect; indirect effect 2, exercise time → mental resilience → mental health, the mediating effect of mental resilience accounted for 20.12% of the total effect; indirect effect 3, exercise time → social support → mental health, the mediating effect of social support accounted for 17.31% of the total effect. Additionally, the upper and lower bounds of the confidence intervals of the direct and indirect effects did not contain 0 (Table 3), showing that exercise predicted mental health directly and through the mediating effects of stress, resilience, and social support. Direct and indirect effects accounted for 35.77 and 64.23% of the total effects, respectively.

**Table 3:** Results of the mediation effect of stress, resilience, and social support.

	**Effect**	**BootSE**	**BootLLCI**	**BootULCI**	**Rate**
Total effect	0.21	0.02	0.17	0.25	100.00%
Direct effect	0.08	0.02	0.04	0.11	35.77%
Indirect effect	0.13	0.02	0.1	0.17	64.23%
Stress	0.06	0.01	0.04	0.07	26.80%
Resilience	0.04	0.01	0.03	0.06	20.12%
Social support	0.04	0.01	0.02	0.05	17.31%

## 4. Discussion

As high working pressure is one of the main causes of occupational sub-health of scientific and technological professionals, this study examined the relationship between physical exercise and mental health among scientific and technical workers, with stress, resilience, and perceived social support as mediators. The results show that exercise predicts mental health directly and through the mediating effects of stress, resilience, and social support. These results emphasize the importance of exercise for the mental health of scientific and technological professionals.

There was a difference in exercise time based on gender, with men spending significantly more time exercising than women. This finding is consistent with previous studies conducted in different cultures, which have shown that men overwhelmingly participate in more intense physical activity than women (10, 32, 33). In addition, there was a difference in exercise times and mental health levels based on age, with workers over 45 years old scoring higher than those 45 years old and younger. Studies have found that science and technology workers often work overtime, lack exercise, work irregular hours, and lack rest, which are likely to cause psychological problems and great mental health risks (1). However, young scientific and technological workers are more prominent (3). Moreover, in terms of educational background, the exercise time and mental health of participants with a PhD were lower than those with master's or bachelor's degrees. We suspect that this may be because they are under greater pressure due to academic activities, such as paper publications and grant applications (3).

The results of this study show that exercise can positively predict mental health, which is consistent with previous studies. Habibzadeh (17) claimed that physical activity can improve general health and happiness. The current study shows that the effect of exercise on the mental health of science and technology professionals is generated through three mediating pathways: stress, resilience, and social support.

Exercise can indirectly affect the mental health of scientific and technological professionals by relieving their stress. As proposed in previous studies (17–19), exercise can be used as an intervention to relieve stress, regulate negative emotions, and promote mental health. In addition, exercise can indirectly affect the mental health of scientific and technological professionals by the mediating effect of resilience. Researchers have found that physical exercise is a protective factor that promotes the development of individual resilience (16). Finally, exercise can indirectly affect the mental health of scientific and technological professionals through social support. This finding is consistent with the results of previous studies. For example, Li (10) found that exercise promoted mental health by providing social support, improving exercise experience, and promoting a series of physiological changes. Social support can provide people with a sense of security, belonging, and self-worth and is an important external resource affecting mental health (24). Exercise can help individuals establish interpersonal relationships and obtain social support, thus achieving the effect of promoting mental health. It is worth noting that stress, resilience, and social support can simultaneously mediate the relationship between exercise and mental health.

### 4.1. Theoretical implications

This study has certain theoretical implications. We explored the mechanisms in the relationship between exercise and the mental health of scientific and technological professionals. Research on exercise and mental health has largely focused on students, and a limited number of scholars have investigated this topic among scientific and technological professionals.

### 4.2. Practical implications

This study also has practical implications. The results highlight the importance of exercise, which implies the necessity of caring for science and technology workers. As the main body of science and technology innovation, the mental health of science and technology workers is of great concern. The results can be helpful for managers in developing effective and reasonable management systems, providing a supportive environment, and thereby improving the mental health level of scientific and technological workers. The relationship between exercise and mental health suggests that exercise may be related to the promotion of mental health among science and technology workers. Exercise can relieve stress, improve psychological resilience, and increase social support, thereby affecting mental health. Therefore, exercise can be used as an effective intervention to improve mental health and promote the improvement of mental health of science and technology workers.

### 4.3. Limitations and future research

This study had some limitations. First, all the scales used in this study were self-report, that may have resulted in self-report bias. A combination of self-report and more objective evaluations (e.g., behavioral, situational) should be adopted in future research to enhance the objectivity of the data. Second, the participants were science and technology workers in a single province, and the sample representativeness was limited, that may not be generalizable to other populations. In future research, data should be collected from different regions of different industries. Finally, the study used a cross-sectional design, so causal relationships could not be determined. Future studies should use a longitudinal design to further explore the causal relationship between exercise and mental health of science and technology professionals. Besides, limited number of variables were studied to explore the relations between exercise and mental health. In future studies, more variables should be considered, and the moderated variables are necessary to be evaluated as well.

## Data availability statement

The raw data supporting the conclusions of this article will be made available by the authors, without undue reservation.

## Ethics statement

The studies involving human participants were reviewed and approved by Henan University Institutional Review Board. All relevant ethical safeguards have been met in relation to patient or subject protection, or animal experimentation. The patients/participants provided their written informed consent to participate in this study.

## Author contributions

YL and KW are the principal investigators for the study, generated the idea, and designed the study. SW and KW were the primary writers of the manuscript and approved all changes. SW, KW, and TD supported the data input and data analysis. TD and KW supported the data collection. All authors were involved in developing, editing, reviewing, and providing feedback for this manuscript and have given approval of the final version to be published.
